# Supplementary material for: The interplay of additivity, dominance, and epistasis on fitness in a diploid yeast cross
Source: Nat Commun. 2022 Mar 18;13:1463. doi: 10.1038/s41467-022-29111-z (PMC8933436; doi:10.1038/s41467-022-29111-z)
Supplement: Supplementary file 1 — Supplementary Information [file 41467_2022_29111_MOESM1_ESM.pdf]

Supplemental information for

The interplay of additivity, dominance, and epistasis on fitness in a diploid yeast cross

Takeshi Matsui, Martin N. Mullis, Kevin Roy, Joseph J. Hale, Rachel Schell, Sasha F. Levy, Ian M. Ehrenreich

Tables

**Drug/chemical**	**Concentration**
Cobalt Chloride	6.675 ug/ml
Copper Sulfate	225 ug/ml
Hydrogen Peroxide	0.006%
Sodium Chloride	225 mM
Rapamycin	0.25 mM
Zeocin	625 ug/ml

Supplementary Table 1. List of chemical additives and their concentrations used in the competition experiments. Chemicals are added to SCM-Ura + 2% glucose (the Glucose1 and Glucose2 growth condition)**.**

Environment	1_rep	2_rep	3_rep	4_rep	total	2+_rep
$CoCl_2$	28858	84334	43211	59599	216002	187177
$CuSO_4$	25020	94378	43159	54555	217112	192096
Glucose1	32545	82656	41473	63861	220535	187990
Glucose2	28985	82262	41400	69888	222535	193550
H_2O_2	29557	86235	44804	57669	218265	188708
NaCl	28581	85554	43625	60870	218630	190050
Rapamycin	25441	87722	40534	65017	218714	193292
Zeocin	25632	82913	44274	64779	217598	191969

Supplementary Table 2. Number of replicate diploid strains in each fitness assay. Column 1 ('Environment') lists the eight pooled fitness assays (including glucose replicates) conducted in this study. Columns 2 - 5 ('1_rep' - '4_rep') list how many diploid strains are present in one, two, three, or four barcoded strain replicates within a given fitness assay. Column 6 ('total') lists the total number of diploid strains present in each fitness assay. Column 7 ('2+_rep') is how many of the total strains are present at least twice in an experiment.

Environment	broad	broad_se	narrow	narrow_se	dom	dom_se	epi	epi_se
$CoCl_2$	0.621	0.011	0.507	0.035	0.012	0.008	0.22	0.038
$CuSO_4$	0.572	0.011	0.453	0.042	0.01	0.008	0.247	0.044
Glucose1	0.745	0.007	0.517	0.025	0.071	0.012	0.147	0.024
Glucose2	0.762	0.007	0.525	0.025	0.076	0.012	0.144	0.024
H_2O_2	0.516	0.015	0.194	0.032	0.061	0.022	0.147	0.055
NaCl	0.553	0.013	0.343	0.037	0.04	0.016	0.156	0.049
Rapamycin	0.576	0.014	0.409	0.034	0.028	0.013	0.2	0.047
Zeocin	0.54	0.015	0.292	0.033	0.036	0.016	0.154	0.053

Supplementary Table 3. Broad and narrow-sense heritability estimates for each fitness assay. Column 1 (‘env’) lists the eight pooled fitness assays (including glucose replicates) conducted in this study. Columns 2 (‘broad’) and 3 (‘broad_se’) list the broad sense heritability estimates and corresponding standard error values. Columns 4 (‘narrow’) and 5 (‘narrow_se’) list the narrow sense heritability estimates and corresponding standard error values. Columns 6 (‘dom’) and 7 (‘dom_se’) list estimates of phenotypic variance explained by dominance and the corresponding standard error values. Columns 8 (‘epi’) and 9 (‘epi_se’) list estimates of phenotypic variance explained by genetic interactions and the corresponding standard error values.

Environment	Number of genotypes	Number of reps per genotype
$CoCl_2$	230474	2.904
$CuSO_4$	229726	2.782
Glucose1	221747	2.613
Glucose2	227025	2.828
H_2O_2	228008	2.814
NaCl	227207	2.782
Rapamycin	230373	2.908
Zeocin	229428	2.91

Supplementary Table 4. The number of unique genotypes and the number of barcode replicates per genotype detected before filtering for quality. Column 1 (‘Environment’) lists the eight pooled fitness assays (including glucose replicates) conducted in this study. Column 2 (‘Number of genotypes’) list the total number of diploid strains detected before filtering for quality. Column 3 (‘Number of rep per genotype’) lists the average number of barcoded strain replicates each diploid was represented before filtering for quality.

Environment	Coefficient of determination	PCR chimera rate
$CoCl_2$	0.695	0.123
$CuSO_4$	0.734	0.167
Glucose1	0.488	0.115
Glucose2	0.558	0.098
H_2O_2	0.593	0.109
NaCl	0.578	0.075
Rapamycin	0.728	0.108
Zeocin	0.611	0.079

Supplementary Table 5. Estimated rate of PCR chimeras. Column 1 (‘Environment’) lists the eight pooled fitness assays (including glucose replicates) conducted in this study. Column 2 (‘Coefficient of determination’) lists the R^2 value when observing the linear relationship between the number of PCR chimeras and the abundance of the involved barcodes: *# of copies of PCR chimera ~ # of copies of barcode1 * # of copies of barcode2*. Column 3 (‘PCR chimera rate’) lists the overall PCR chimera rate estimated using this linear model.

Supplementary figures

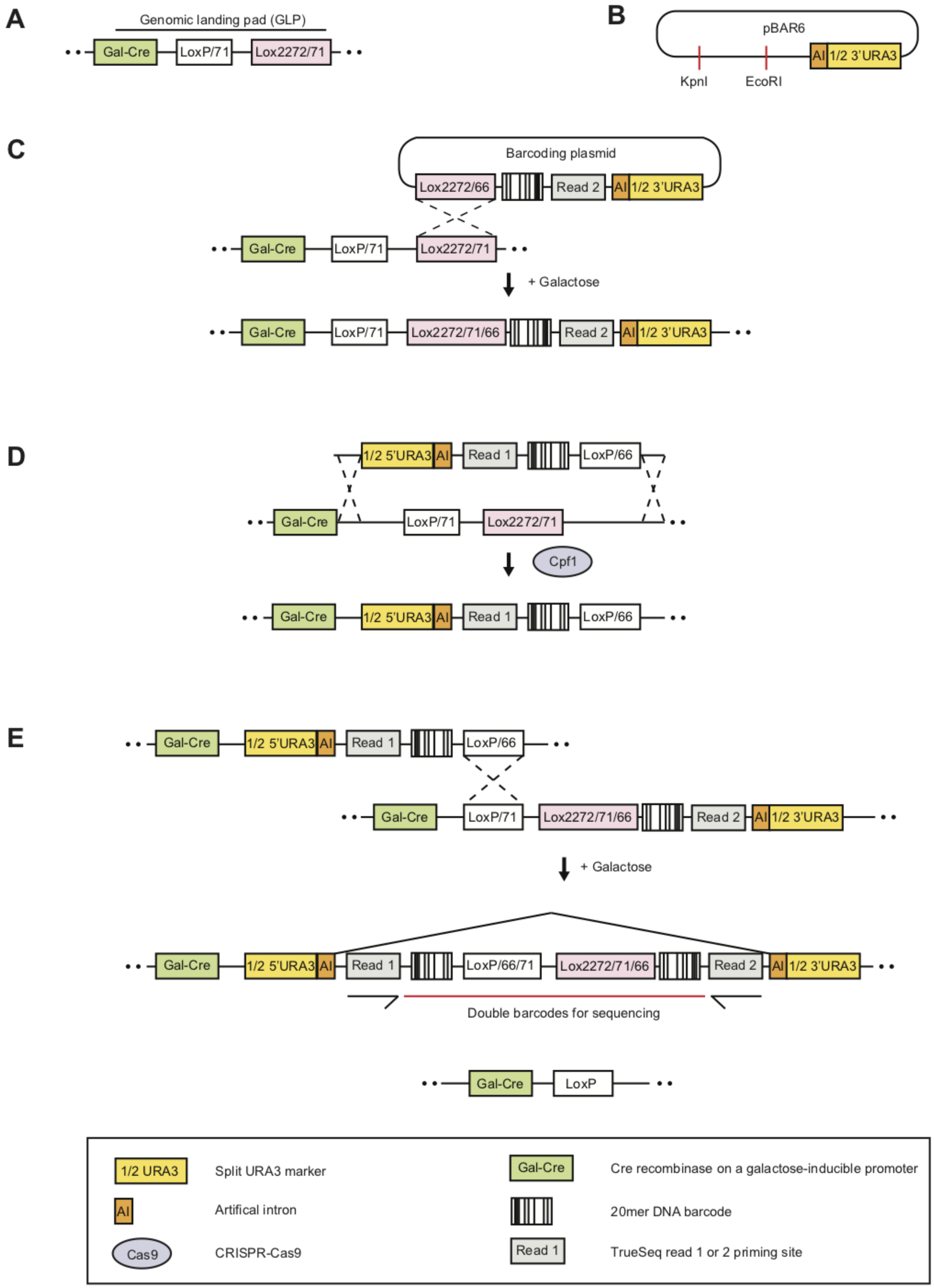

Supplementary Figure 1. Workflow of how haploid and diploid segregants were barcoded. A. Each parental strain was engineered to have a genomic “landing pad” containing two partially crippled LoxP sites, LoxP/71 and Lox2272/71, and a galactose-inducible Cre recombinase at the *YBR209W* locus. **B.** pBAR6 plasmid used to make the library of barcoding plasmids for MATα segregants. **C.** Barcoding plasmids were made by combining linearized pBAR6 with a PCR product containing a partially crippled Lox2272/66 site, a random 20-mer barcode sequence, and a partial TrueSeq read 2 adapter sequence using Gibson assembly. The barcoding plasmids were then individually transformed into each MATα segregant and integrated into the genome at the genomic “landing pad” using cre-recombinase mediated homologous recombination at the Lox2272 site. **D.** MAT**a** segregants were barcoded by integrating a PCR product containing a split URA3 marker, a partial TruSeq read1 adapter sequence, a random 20-mer barcode, and a partially crippled LoxP/66 at the genomic “landing pad” using CRISPR/Cas9 mediated homologous recombination. **E.** After the *MAT**a*** and *MATα* segregants were mated, the two barcodes were brought onto the same chromosome using site-directed chromosomal translocation via Cre-LoxP homologous recombination.

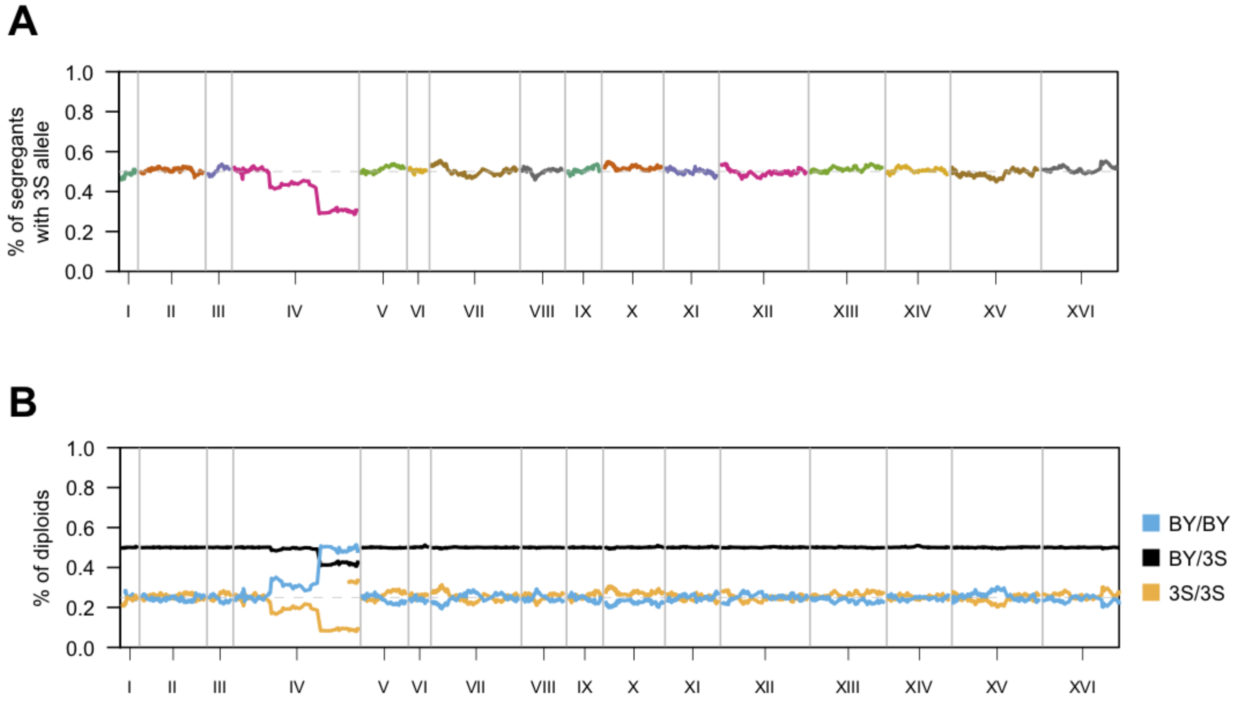

Supplementary Figure 2. Genome-wide allele frequencies of haploid segregants and the inferred genome-wide allele frequencies of diploid segregants. A. Genome-wide allele frequencies of haploid BYx3S segregants. With the exception of a region on Chromosome IV, allele frequencies were balanced in the BYx3S segregants used to generate the diploid population. **B.** Inferred genome-wide allele frequencies of diploid BYx3S strains. Genotypes for diploid strains were generated *in silico* using sequencing data from segregants containing the barcodes present in each diploid.

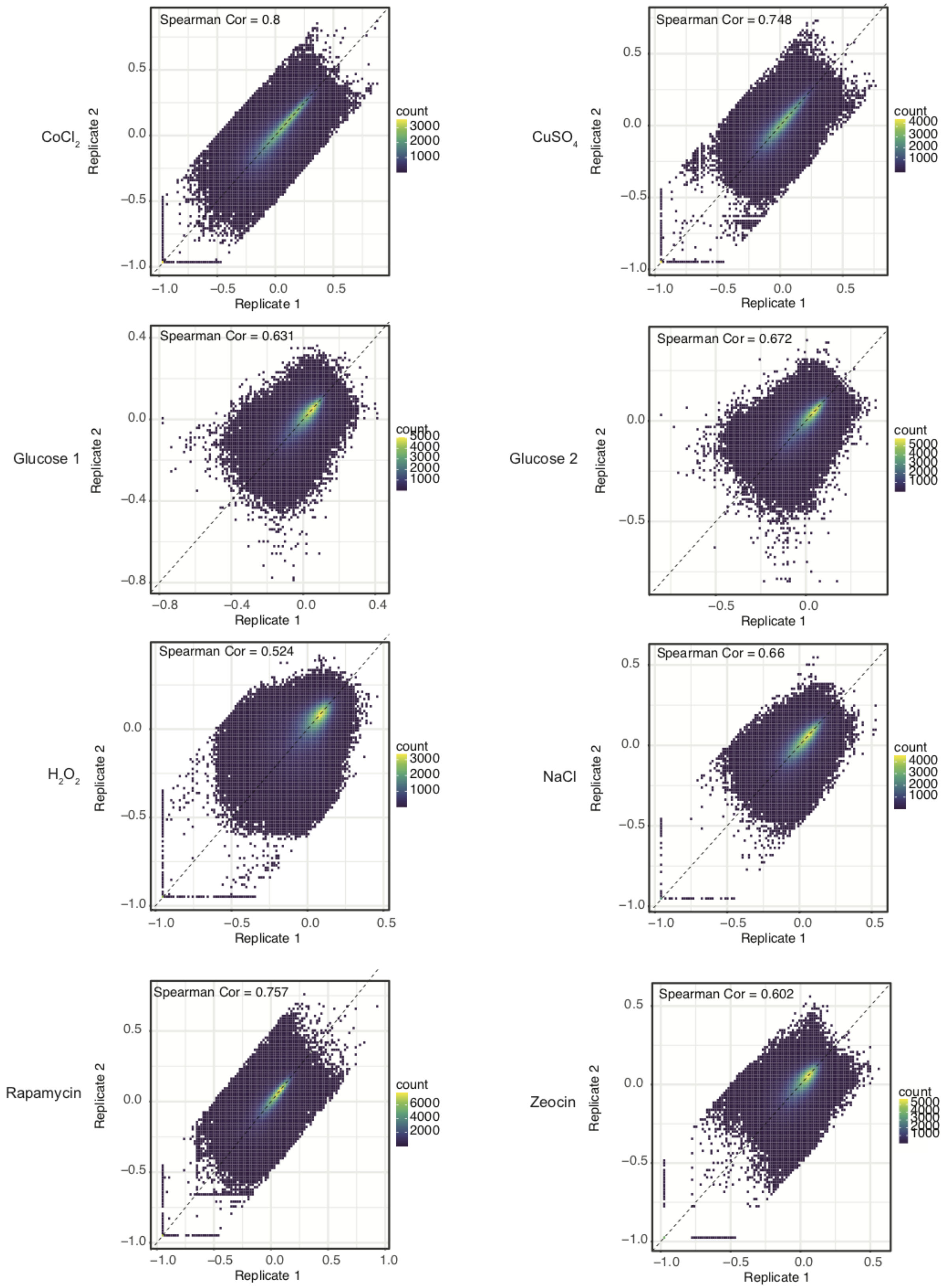

CoCl2
Spearman Cor = 0.8
CuSO4
Spearman Cor = 0.748
Glucose 1
Spearman Cor = 0.631
Glucose 2
Spearman Cor = 0.672
H2O2
Spearman Cor = 0.524
NaCl
Spearman Cor = 0.66
Rapamycin
Spearman Cor = 0.757
Zeocin
Spearman Cor = 0.602
Replicate 1
Replicate 2
count

Supplementary Figure 3. Correlation of fitness estimates between strain replicates in each experiment. Between two and four barcoded replicates of all strains were included in each pooled fitness assay. Fitness estimates between strain replicates were well correlated ($0.524 \leq$ Spearman's $\rho \leq 0.8$) within each experiment.

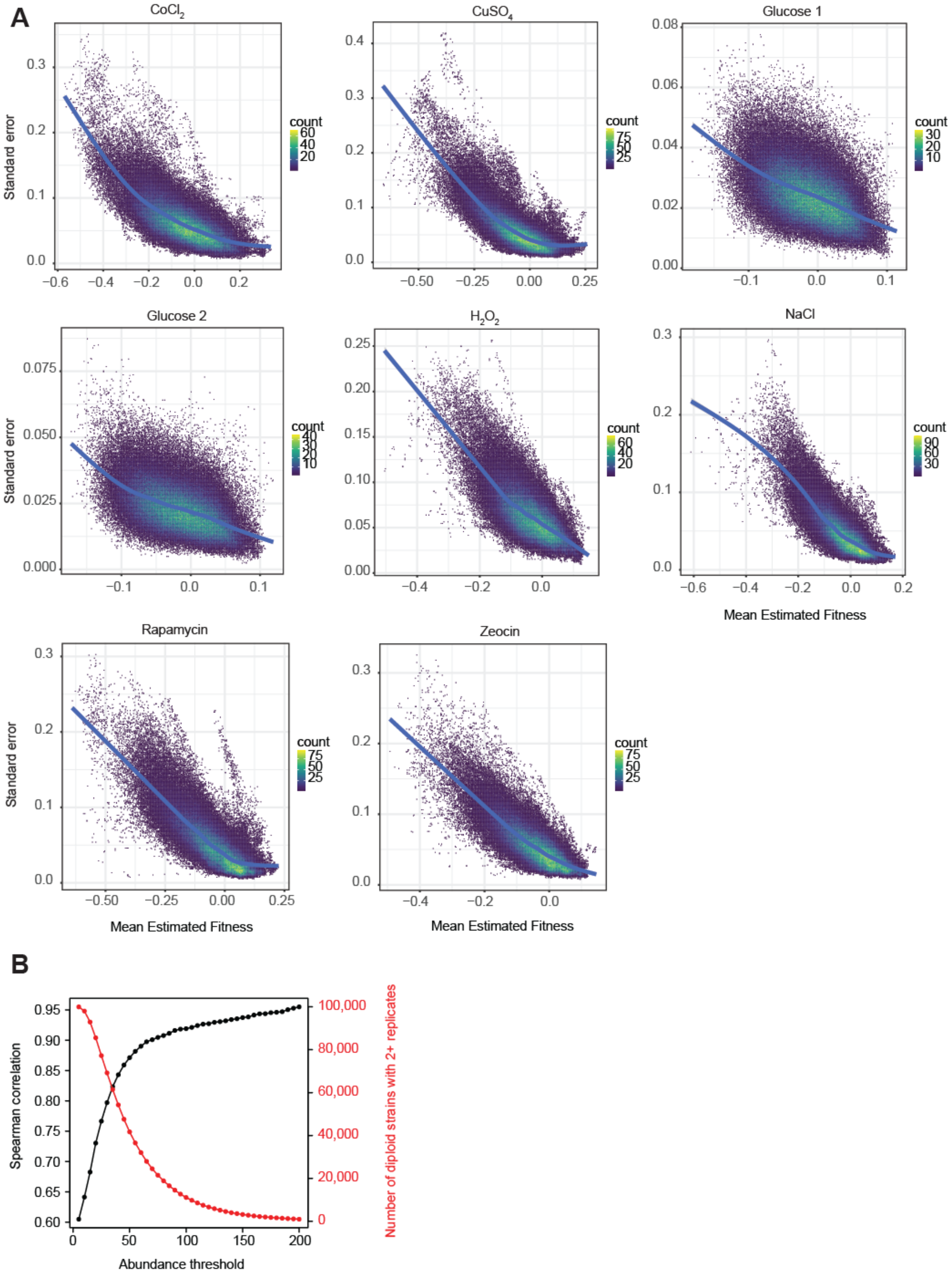

A
$CoCl_2$
$CuSO_4$
Glucose 1
Glucose 2
H_2O_2
NaCl
Rapamycin
Zeocin
Standard error
Mean Estimated Fitness
count
B
Spearman correlation
Number of diploid strains with 2+ replicates
Abundance threshold
0.60
0.65
0.70
0.75
0.80
0.85
0.90
0.95
0
20,000
40,000
60,000
80,000
100,000
0
50
100
150
200

Supplementary Figure 4. Accuracy of fitness estimates (measurement noise) is significantly impacted by the fitness and initial frequency of a diploid strain. A. A negative relationship was observed between the mean fitness of replicates and the standard error of the mean. Diploid strains with the lowest fitnesses had the highest standard errors in fitness estimates between replicates while strains with the highest fitness had the lowest measurement noise. **B.** Examination of how the correlation of fitness between replicate strains changes if we only include replicate strains that are abundant in the pool. Correlation of fitness between replicates improved significantly as the required abundance threshold (double barcode count at the first time point) was increased.

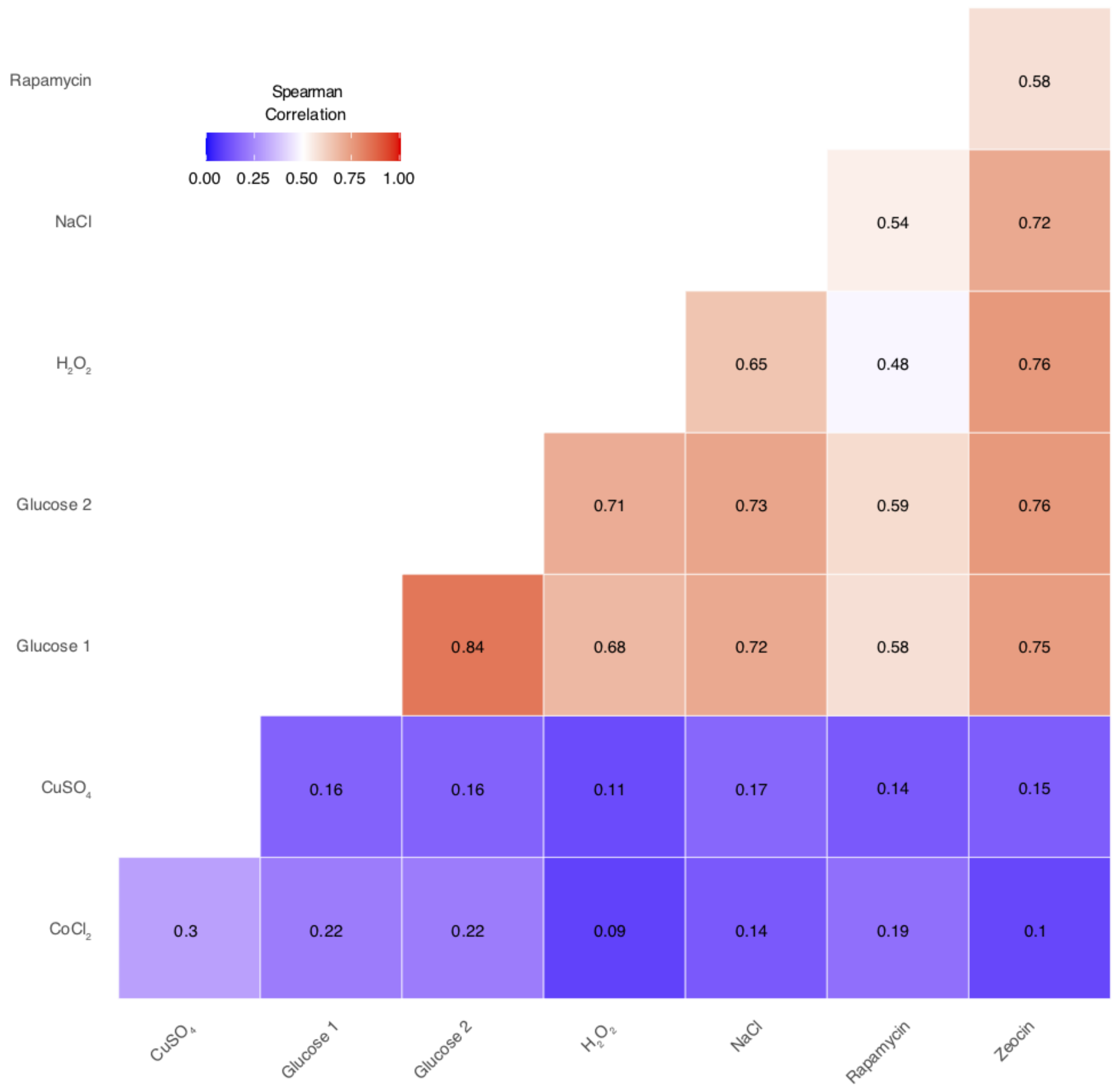

Supplementary Figure 5. Correlation of fitness across environments. Cells in the heatmap contain Spearman's correlation coefficients of the mean fitnesses of strains across all environments. Two environments, $CoCl_2$ and $CuSO_4$, were less correlated with other environments and each other.

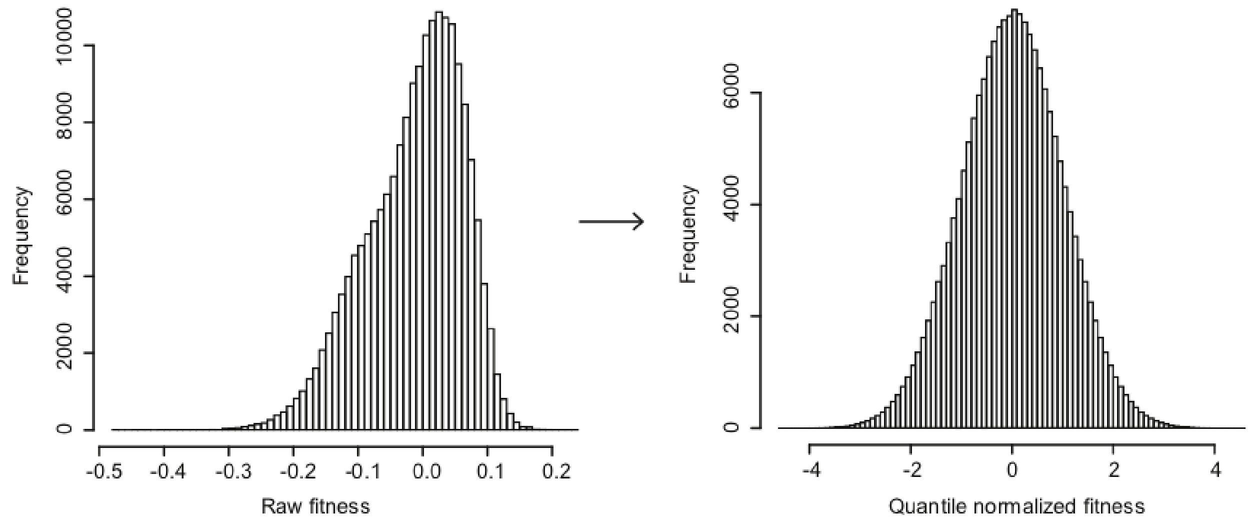

Supplementary Figure 6. Quantile normalization of fitnesses. Because the distributions of fitness were slightly left skewed, mean fitnesses were quantile normalized. This quantile normalized fitness estimate was used as the *fitness* phenotype for all downstream analyses.

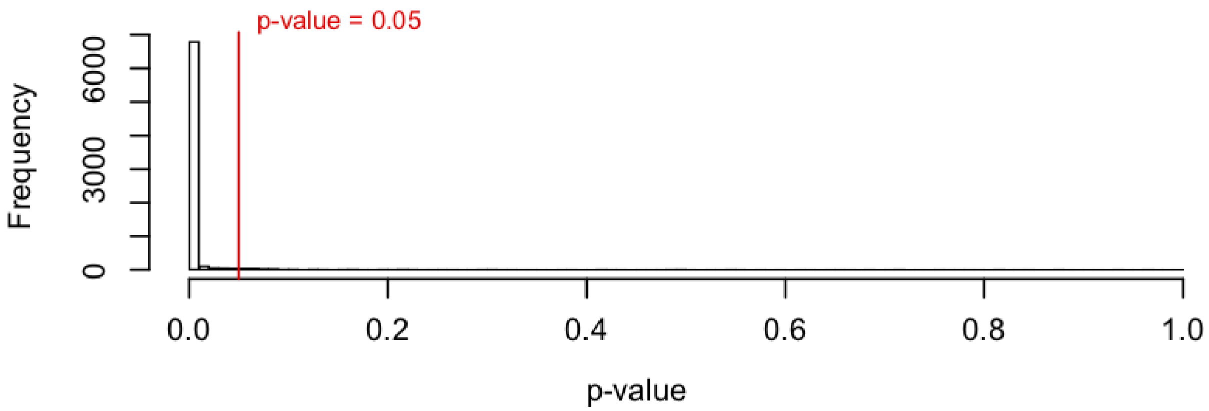

Supplementary Figure 7. Most loci are detected as significant without family correction. Histogram showing the p-values of all 7,742 SNP markers when tested for significance using a fixed effects linear model without correcting for family structure. The p-values shown in this plot are not corrected for multiple testing. The red line shows the nominal significance threshold at p-value = 0.05.

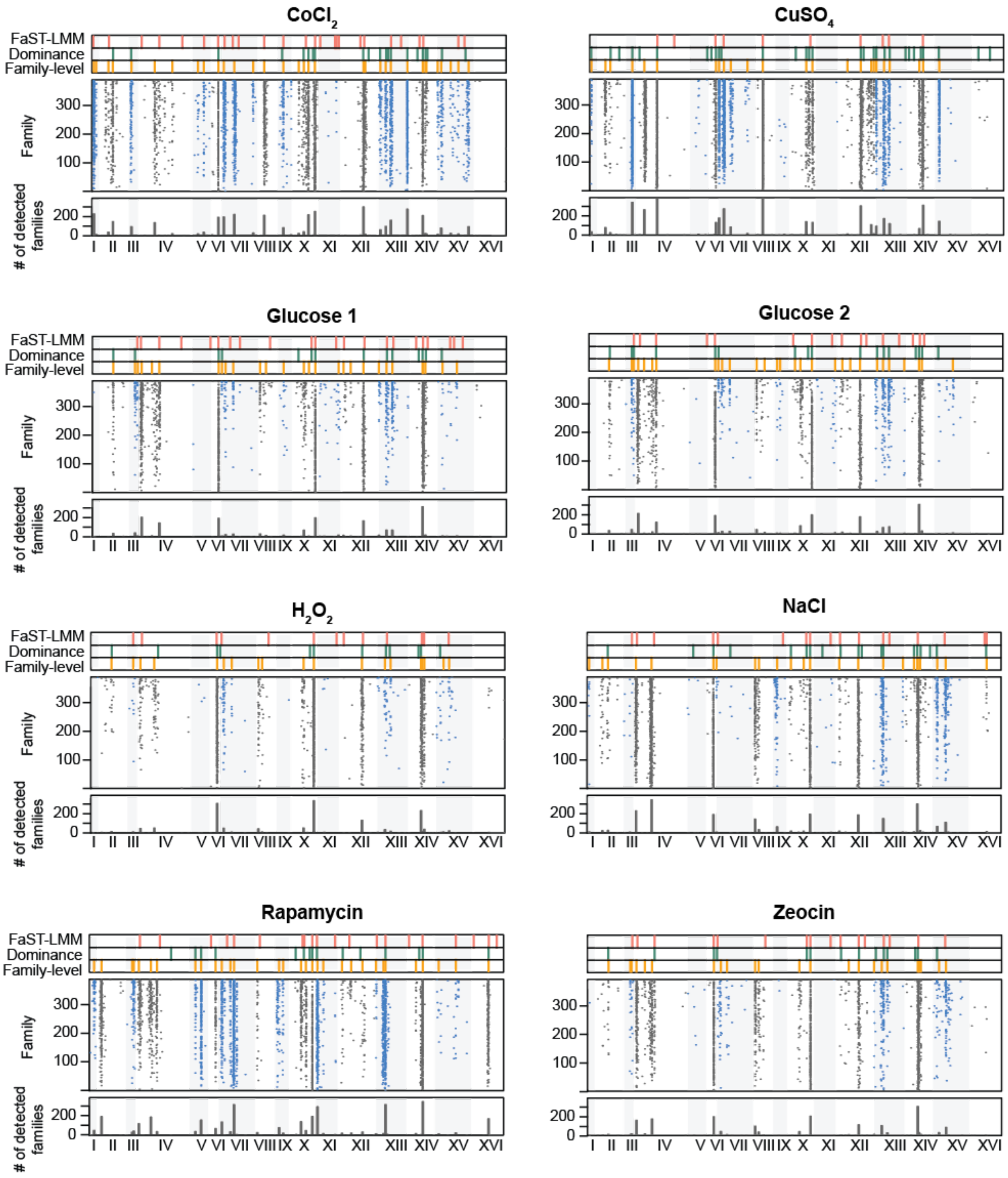

CoCl$_2$
CuSO$_4$
Glucose 1
Glucose 2
H$_2$O$_2$
NaCl
Rapamycin
Zeocin
FaST-LMM
Dominance
Family-level
Family
# of detected families
300
200
100
200
0
I II III IV V VI VII VIII IX X XI XII XIII XIV XV XVI

Supplementary Figure 8. Loci with individual effects on fitness in each environment. Panels from top to bottom are 1) loci detected by the mixed effects linear model FaST-LMM (red bars), 2) dominance loci detected by the fixed effect linear model using the non-additive portions of each diploid's phenotype (green bars), 3) loci enriched for detection in family-level scans (orange bars) 4) loci detected using family-level scans (black or blue points), where each row is a different *MAT**a*** family, and 5) the total number of detections across families for each 20 kb interval (grey bars).

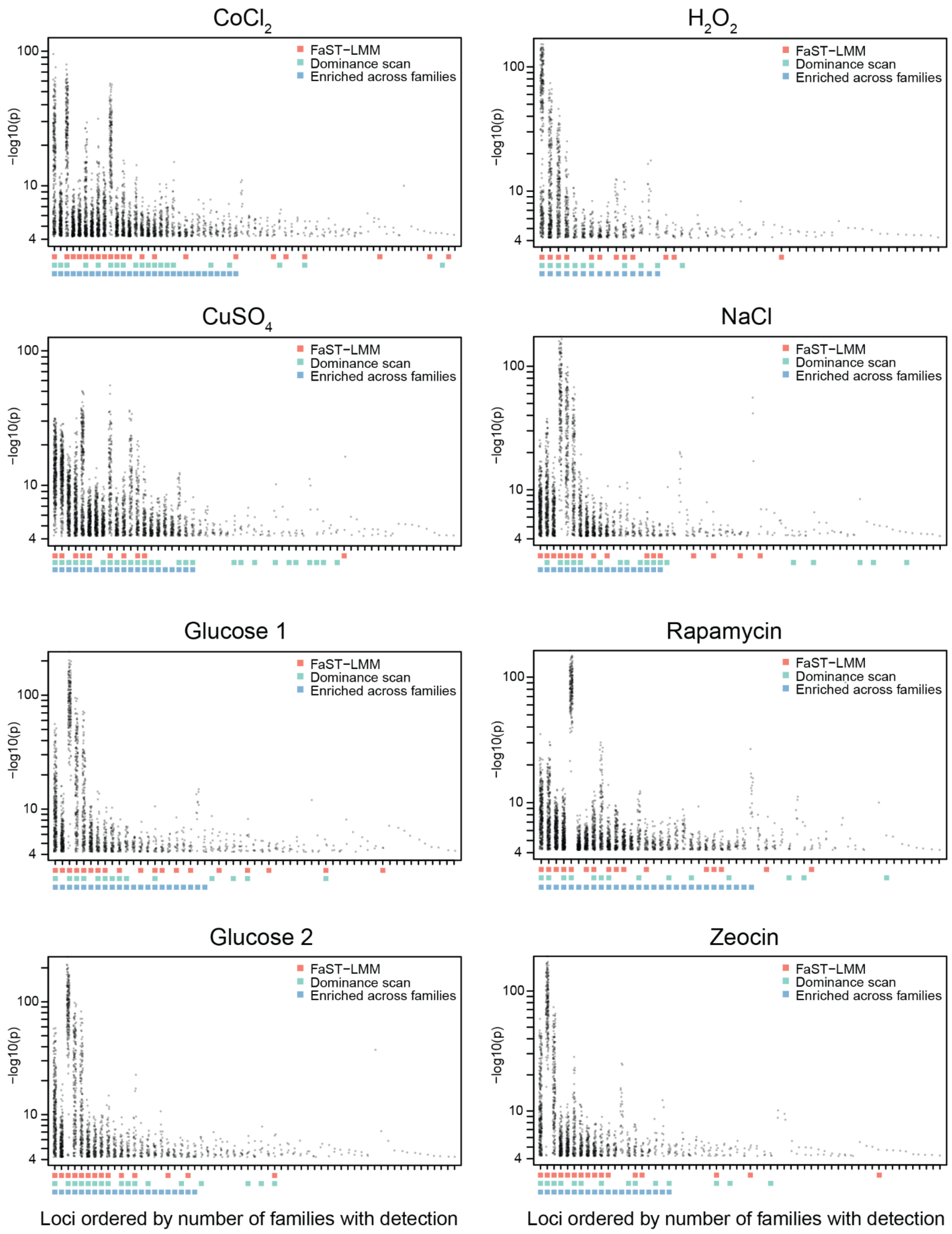

$CoCl_2$
H_2O_2
$CuSO_4$
NaCl
Glucose 1
Rapamycin
Glucose 2
Zeocin
FaST−LMM
Dominance scan
Enriched across families
−log10(p)
Loci ordered by number of families with detection

Supplementary Figure 9. Distinct loci show substantial variation in detection across families. Genome-wide mapping was conducted in each family using a fixed effects linear model *fitness ~ locus*, where the *fitness* term corresponds to the vector of quantile normalized fitness values of individuals within a family and the *locus* term corresponds to the vector of these individuals' genotypes at a given marker. To determine appropriate significance thresholds, 1,000 permutations were conducted with the correspondence between genotypes and phenotypes randomly shuffled each time. Only p-values from family scans that were significant after multiple testing corrections are shown, with a $\log_{10}$ scaling employed. Red, green, and blue labels denote distinct loci in family-level scans that were identified by FaST-LMM, dominance scans, or enrichment tests, respectively.

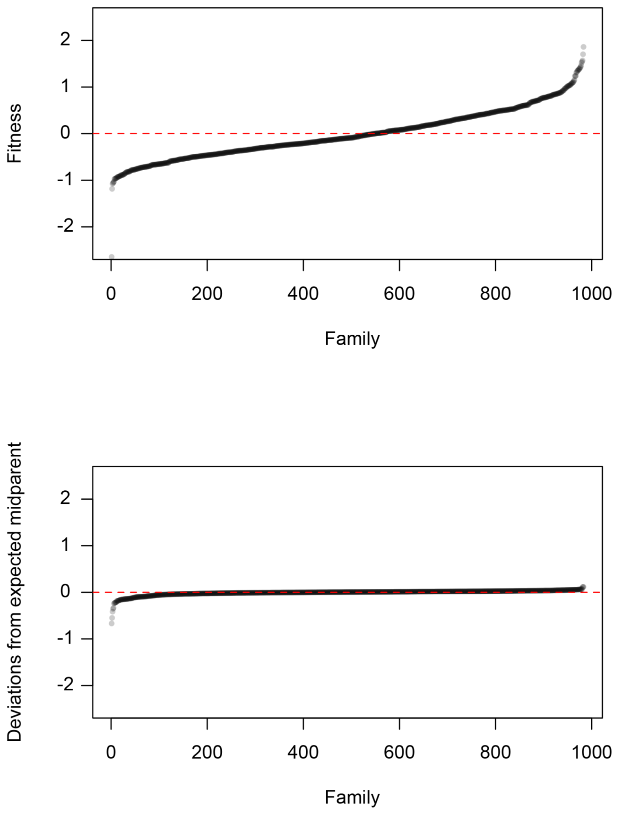

Supplementary Figure 10. Accounting for differences in mean parental fitness corrects for family-level fitness effects in the population. A. The mean fitness of each of the 392 *MAT**a*** and 591 *MATα* families in glucose, ordered from lowest to highest mean fitness. **B.** The mean *residuals*, or the non-additive portions of each diploid's phenotype, for each family after accounting for mean parental fitness.

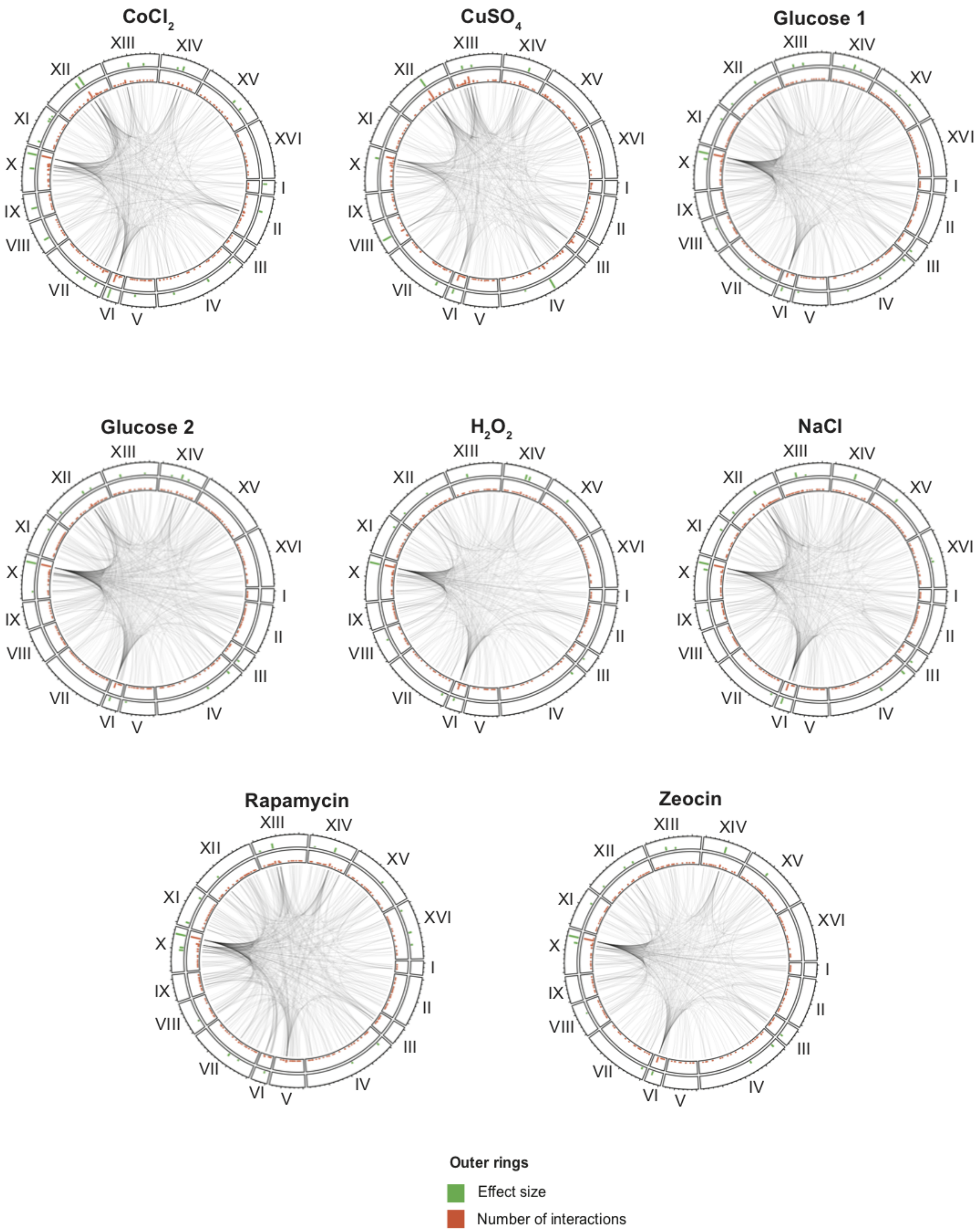

$CoCl_2$
$CuSO_4$
Glucose 1
Glucose 2
H_2O_2
NaCl
Rapamycin
Zeocin
I
II
III
IV
V
VI
VII
VIII
IX
X
XI
XII
XIII
XIV
XV
XVI
Outer rings
Effect size
Number of interactions

Supplementary Figure 11. Pairwise genetic interactions detected in each environment. In each plot, interior lines connect regions of the genome involved in pairwise interactions. Outer rings contain barplots with height corresponding to the number of pairwise interactions at each locus (orange) and its absolute effect size (green) in the entire population.

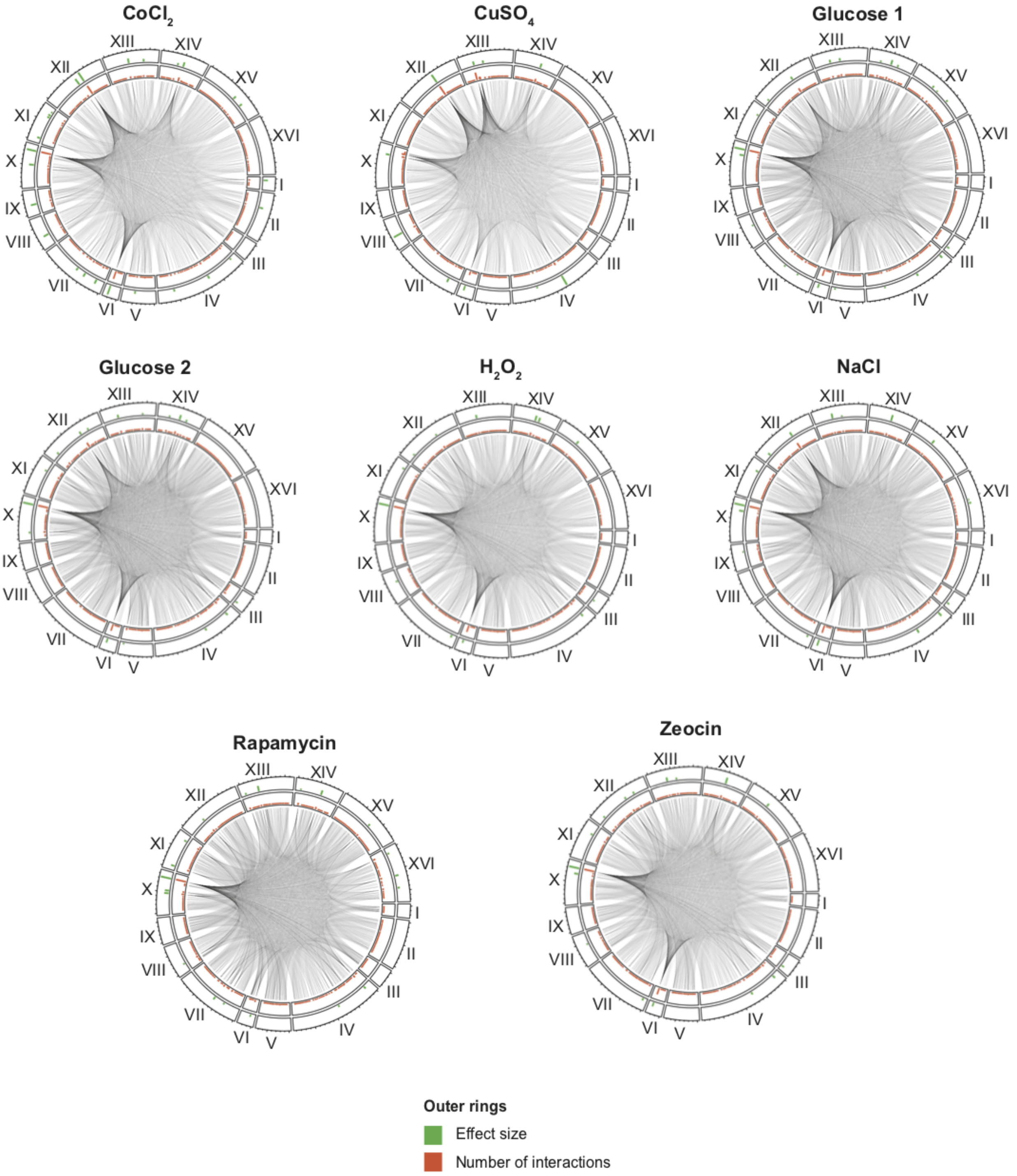
CoCl2
CuSO4
Glucose 1
Glucose 2
H2O2
NaCl
Rapamycin
Zeocin
I II III IV V VI VII VIII IX X XI XII XIII XIV XV XVI
Outer rings
Effect size
Number of interactions

Supplementary Figure 12. Three-way genetic interactions detected in each environment. In each plot, interior lines connect regions of the genome involved in three-way interactions. Outer rings contain barplots with height corresponding to the number of three-way interactions at each locus (orange) and its absolute effect size (green) in the entire population.

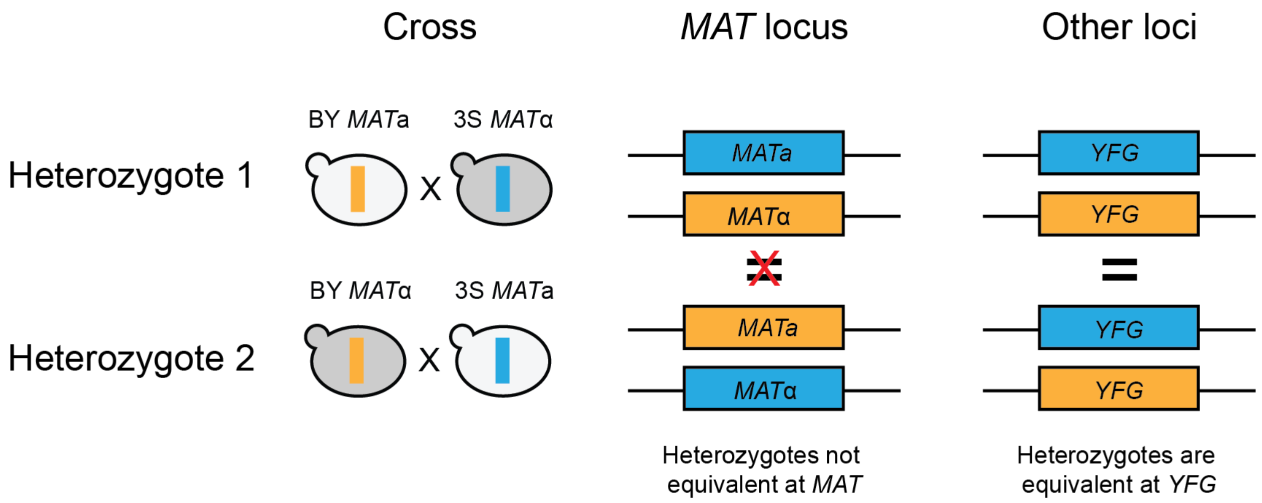

Supplementary Figure 13. Genetic variation at the mating locus results in distinct heterozygote classes. To ensure segregation of the mating locus, both BY *MAT**a*** x 3S *MATα* and 3S *MAT**a*** x BY *MATα* crosses were performed using isogenic strains that had been mating type switched. Pairwise matings between these haploids results in two possible heterozygous genotypes at the mating locus in the resulting diploids: BY *MAT**a*** / 3S *MATα* and 3S *MAT**a*** / BY *MATα*.
