# Supplementary material for: The interplay of additivity, dominance, and epistasis on fitness in a diploid yeast cross
Source: Nat Commun. 2022 Mar 18;13:1463. doi: 10.1038/s41467-022-29111-z (PMC8933436; doi:10.1038/s41467-022-29111-z)
Supplement: Supplementary file 3 — Reporting Summary [file 41467_2022_29111_MOESM3_ESM.pdf]

nature portfolio

Corresponding author(s): Ian M. Ehrenreich, Sasha F. Levy

Last updated by author(s): January 27, 2022

Reporting Summary

Nature Portfolio wishes to improve the reproducibility of the work that we publish. This form provides structure for consistency and transparency in reporting. For further information on Nature Portfolio policies, see our Editorial Policies and the Editorial Policy Checklist.

Statistics

For all statistical analyses, confirm that the following items are present in the figure legend, table legend, main text, or Methods section.

n/a	Confirmed	
☐	☒	The exact sample size (*n*) for each experimental group/condition, given as a discrete number and unit of measurement
☐	☒	A statement on whether measurements were taken from distinct samples or whether the same sample was measured repeatedly
☐	☒	The statistical test(s) used AND whether they are one- or two-sided<br>*Only common tests should be described solely by name; describe more complex techniques in the Methods section.*
☐	☒	A description of all covariates tested
☐	☒	A description of any assumptions or corrections, such as tests of normality and adjustment for multiple comparisons
☐	☒	A full description of the statistical parameters including central tendency (e.g. means) or other basic estimates (e.g. regression coefficient) AND variation (e.g. standard deviation) or associated estimates of uncertainty (e.g. confidence intervals)
☐	☒	For null hypothesis testing, the test statistic (e.g. *F*, *t*, *r*) with confidence intervals, effect sizes, degrees of freedom and *P* value noted<br>*Give P values as exact values whenever suitable.*
☒	☐	For Bayesian analysis, information on the choice of priors and Markov chain Monte Carlo settings
☐	☒	For hierarchical and complex designs, identification of the appropriate level for tests and full reporting of outcomes
☐	☒	Estimates of effect sizes (e.g. Cohen's *d*, Pearson's *r*), indicating how they were calculated

Our web collection on statistics for biologists contains articles on many of the points above.

Software and code

Policy information about availability of computer code

Data collection	No software was used in the collection of data for this manuscript.
Data analysis	Sequencing data was aligned using BWA (ver 0.7.17-r1188). Sequencing data was processed using SAMtools (ver 0.19-4428cd). Barcode cluster analysis was performed using Bartender (ver 1.1), available at https://github.com/LaoZZZZZ/bartender-1.1. Fitness estimates were generated using PyFitSeq (ver 1), available at https//github.com/FangfeiLi05/PyfitSeq. Data analysis was performed using R (ver 4.0.2) and Python (ver 3.7.6). Vector of initial genotype calls was corrected with a Hidden Markov Model (HMM), implemented using the HMM package version 1.0 in R. Broad and narrow sense heritabilities were estimated using the Sommer package ver 4.1.5 in R. Fitness estimates were quantile normalized such that the data is normally distributed using the bestNormalize ver 1.8.2 in R. The genotype table was reformatted as a binary biallelic genotype table (BED) using PLINK ver 1.07. Genome-wide scan for one-locus effects were conducted using Factored Spectrally Transformed Linear Mixed Models (FaST-LMM) ver 0.5.5, available at https://github.com/fastlmm/FaST-LMM. Custom scripts used in this study are available at: https://github.com/tmatsui2/Matsui-et-al.-2021-Supplemental_information.git.

For manuscripts utilizing custom algorithms or software that are central to the research but not yet described in published literature, software must be made available to editors and reviewers. We strongly encourage code deposition in a community repository (e.g. GitHub). See the Nature Portfolio guidelines for submitting code & software for further information.

Data

Policy information about availability of data

All manuscripts must include a data availability statement. This statement should provide the following information, where applicable:

- Accession codes, unique identifiers, or web links for publicly available datasets
- A description of any restrictions on data availability
- For clinical datasets or third party data, please ensure that the statement adheres to our policy

All data generated or analysed during this study are included in this published article (and its supplementary information files). A Data Availability statement is included in the manuscript. Raw barcode sequencing data are available from the NCBI Sequence Read Archive as accession PRJNA781980. Additional data (Supplementary data SD1-11) are available in Mendeley data (https://data.mendeley.com/datasets/96ghpptzvf). S288C reference genome ver R9-1-1 was used in this study and is available at http://sgd-archive.yeastgenome.org/sequence/S288C_reference/genome_releases/.

Field-specific reporting

Please select the one below that is the best fit for your research. If you are not sure, read the appropriate sections before making your selection.

☒ Life sciences ☐ Behavioural & social sciences ☐ Ecological, evolutionary & environmental sciences

For a reference copy of the document with all sections, see nature.com/documents/nr-reporting-summary-flat.pdf

Life sciences study design

All studies must disclose on these points even when the disclosure is negative.

Sample size	Our sample size is the largest linkage mapping study in S. cerevisiae to date. The sample size was designed to be as large as possible, so as to provide the highest possible statistical power.
Data exclusions	Diploid strains with only a single barcoded replicate were excluded from analyses, as there was no way to assess the reproducibility of these fitness estimates.
Replication	All included diploid strains had multiple barcode replicates. Replicate fitness measurements from different barcodes in the same environment generally showed high correlations. We also replicated one condition (glucose) twice, with the experiments showing high concordance. All other conditions were only performed once.
Randomization	All strains were used in all experiments, so no randomization was necessary.
Blinding	There were no a priori experimental groupings in this study, so no blinding was needed.

Reporting for specific materials, systems and methods

We require information from authors about some types of materials, experimental systems and methods used in many studies. Here, indicate whether each material, system or method listed is relevant to your study. If you are not sure if a list item applies to your research, read the appropriate section before selecting a response.

Materials & experimental systems

n/a	Involved in the study	
☒	☐	Antibodies
☐	☒	Eukaryotic cell lines
☒	☐	Palaeontology and archaeology
☒	☐	Animals and other organisms
☒	☐	Human research participants
☒	☐	Clinical data
☒	☐	Dual use research of concern

Methods

n/a	Involved in the study	
☒	☐	ChIP-seq
☒	☐	Flow cytometry
☒	☐	MRI-based neuroimaging

Eukaryotic cell lines

Policy information about cell lines

Cell line source(s)	The lab strain BY4716 is a haploid derivative of the commonly used Saccharomyces cerevisiae reference strain S288C. 3221343S is a clinical isolate of S. cerevisiae. Both strains were readily available in the Ehrenreich lab.
Authentication	Strains were authenticated by analysis of whole genome sequencing compared against the S288C genome.

Mycoplasma contamination	Cell lines were not tested for Mycoplasma contamination.
Commonly misidentified lines (See ICLAC register)	*Name any commonly misidentified cell lines used in the study and provide a rationale for their use.*
